# Repercussions of the pandemic on tuberculosis control actions from the perspective of health professionals

**DOI:** 10.1590/0034-7167-2023-0477

**Published:** 2024-12-13

**Authors:** Sabrina da Silva de Souza, Roxana Isabel Cardozo Gonzales, Hellen Cristina Sthal, Daiane Cardoso da Silva, Marcela Flavia Lopes Barbosa, Tatiana Conceição

**Affiliations:** I Universidade Federal de Santa Catarina. Florianópolis, Santa Catarina, Brazil; II Universidade Federal de Goiás. Goiânia, Goiás, Brazil

**Keywords:** Tuberculosis, Primary Health Care, COVID-19, Health Services Research, Workforce., Tuberculosis, Atención Primaria de Salud, COVID-19, Evaluación en Salud, Recursos Humanos.

## Abstract

**Objectives:**

to analyze the repercussions of the COVID-19 pandemic on tuberculosis control actions from the perspective of primary health care professionals.

**Methods:**

this descriptive study with a qualitative approach was conducted from November 2022 to April 2023, using semi-structured interviews with 11 key informant professionals from primary health care units in a Brazilian capital. Data were organized using Atlas.ti 22.0 software and subjected to thematic-categorical content analysis.

**Results:**

the pandemic scenario caused alterations in the work process, necessitating abrupt adaptations, and led to detrimental impacts on the health of professionals and tuberculosis control actions, which were reduced or discontinued.

**Final Considerations:**

there was evident unpreparedness and a lack of resources from various governmental levels and health services to handle the public health emergency situation without severe harm to the provision of essential services.

## RESUMEN

**Objetivos:**

analizar las repercusiones de la pandemia de COVID-19 en las acciones de control de la tuberculosis, desde la perspectiva de los profesionales de salud de la Atención Primaria de Salud.

**Métodos:**

estudio descriptivo, con enfoque cualitativo, realizado en el período de noviembre de 2022 a abril de 2023, mediante entrevistas semiestructuradas con 11 profesionales informantes clave de unidades básicas de salud de una capital brasileña. Los datos fueron organizados en el software Atlas.ti 22.0 y sometidos a análisis de contenido temático-categorial.

**Resultados:**

el escenario pandémico provocó alteraciones en el proceso de trabajo, con necesidad de adaptaciones abruptas, y causó perjuicios en la salud de los profesionales y en las acciones de control de la tuberculosis, que fueron reducidas o descontinuadas.

**Consideraciones Finales:**

se evidenció la falta de preparación y recursos de las diversas instancias gubernamentales y de los servicios de salud para enfrentar la situación de emergencia en salud pública, sin que hubiera graves perjuicios en la oferta de servicios esenciales.

## INTRODUCTION

Tuberculosis (TB) is a highly contagious disease that remains a severe global public health problem, especially in developing countries. In 2022, 10.6 million people worldwide contracted TB, and approximately 1.3 million died from the disease^(1)^. In the Americas, Brazil bears the highest burden of TB (33%)^(2)^ and is on the World Health Organization’s (WHO) list of priority countries for 2021-2025 due to its high TB burden and high TB/HIV coinfection rate^(1)^. In 2022, around 78,000 people in Brazil contracted TB, and approximately 5,000 died from the disease^(3)^.

Due to the epidemiological, economic, and social magnitude of TB, its elimination as a public health problem is included in the Sustainable Development Goals (SDGs), established by the United Nations (UN) in 2015. In the same year, the WHO launched the global End TB strategy, aiming for a 90% reduction in incidence and a 95% reduction in the mortality rate from the disease by 2035^(4)^. In 2017, Brazil published the National Plan for Ending Tuberculosis as a Public Health Problem^(5)^. However, the goals of the first phase of the plan (2017-2020) were not achieved, and the health and social crisis exacerbated by the COVID-19 pandemic made achieving the goals of the subsequent phases even more complex and distant^(2)^.

In 2020, the first year of the pandemic, it is estimated that there was an 18% reduction in global TB case detection compared to 2019^(3,6)^. In Brazil, there was a 12.1% reduction in the disease’s incidence rate, dropping from 37.9 cases per 100,000 inhabitants in 2019 to 33.3 in 2020. In 2021, there were 34.9 cases per 100,000 inhabitants, and in 2022, 36.3, indicating a partial recovery in detection, though still below pre-pandemic levels^(3)^.

In 2019, the TB treatment interruption rate was 12%, increasing to 12.7% in 2020 and 14.6% in 2021^(3,7,8)^. There was a 0.8% increase in the number of deaths between 2019 and 2020. In 2021, the increase was 12.0% compared to 2019^(3)^. It is evident that with the advance of COVID-19 in 2020 and 2021, TB indicators worsened significantly.

Brazilian health services experienced an explosive increase in demand, surpassing their structural capacity, including in Primary Health Care (PHC), which plays a crucial role in TB prevention, surveillance, detection, and treatment actions. The reduction in the quantity and quality of these control actions results in delayed diagnoses, inadequate treatments, and increased spread in communities, which can lead to high costs for health systems and, especially, for the lives of affected individuals and families^(6)^, further worsening the country’s epidemiological profile^(2)^.

Despite the importance of PHC in controlling TB and the significant impacts generated by the pandemic, scientific publications on the subject remain scarce, particularly from the perspective of social actors (health professionals) who worked in PHC during this period. Therefore, it is essential to highlight these health professionals and the challenges they faced during the pandemic, reflecting on the work process in PHC, the care and attention given to people with TB, and the relevance of the health workforce in reorganizing disease control actions post-pandemic.

## OBJECTIVES

To analyze the repercussions of the COVID-19 pandemic on tuberculosis control actions from the perspective of primary health care professionals.

## METHODS

### Ethical Aspects

The research was approved by the Research Ethics Committee (CEP) of the University Hospital of the Federal University of Goiás, the proposing institution of the Matrix project. To ensure the confidentiality of the participants’ identities, they were identified by the letter “E” followed by a numerical order (from E1 to E11). All participants were informed about the objectives and procedures of the research and were interviewed only after reading and signing the Informed Consent Form (ICF).

### Study Type

This is a descriptive study with a qualitative approach^(9)^, part of the multicenter mixed-method research titled “Repercussion of the COVID-19 pandemic on tuberculosis in Brazilian capitals: reality and new perspectives in Primary Health Care,” which is being developed in six Brazilian capitals (Florianópolis, Goiânia, João Pessoa, Maceió, Porto Velho, and São Paulo), funded by the National Council for Scientific and Technological Development (CNPq) - Call No. 18/2021. The recommendations of the Consolidated Criteria for Reporting Qualitative Research (COREQ)^(10)^ were followed.

### Study Setting

The study was conducted in PHC units in Florianópolis, the capital of the state of Santa Catarina, in the southern region of Brazil. The municipality has 51 basic health units, divided into four districts: mainland, center, north, and south^(11)^, and achieved 76.75% basic care coverage in 2020^(12)^. In 2022, the TB incidence rate was 33 new cases per 100,000 inhabitants^(3)^. Care for this disease in the municipality is decentralized and provided in all health units.

### Data Source

The study included health professionals identified as key informants (by the manager or the team) from PHC units selected through simple random sampling from the sample of units that comprised the quantitative phase of the matrix research. They were invited via phone, email, or WhatsApp.

Inclusion criteria included being active in PHC services for at least three months during the pandemic period. Professionals who were absent from their activities for any reason during the data collection period were excluded. The sample^(13)^, composed of 11 key informants, was defined using the data saturation technique^(14)^. Saturation was evaluated and discussed by two researchers from the team. There were no refusals or withdrawals from participation.

### Data Collection and Organization

Data collection was conducted from November 2022 to April 2023 through semi-structured interviews^(13)^ based on a script developed by the researchers. The script included identification data (such as gender and age), professional data (profession and length of time working in PHC and at the unit), and open-ended questions about the professional experience during the COVID-19 context, the provision of TB control actions, and the repercussions of the pandemic on these actions. The script was reviewed by two specialists and tested with three nursing professionals who were not part of the sample to assess the clarity of the interview guiding questions.

The interviews were conducted in a reserved room within the health units, scheduled according to the health professional’s preference and availability. Each participant was interviewed only once. All interviews were conducted by nurse researchers who were part of the team (faculty and postgraduate students) and who were adequately trained. There was no prior relationship established with the participants before the start of the research. The researchers and the research objectives and procedures were presented to the participants at the time of the invitation to participate.

A field diary was used to record occurrences and observations made by the researchers. Field notes were recorded immediately after each interview. The interviews lasted an average of 10 minutes, were recorded using a voice recorder, transcribed in full by the researchers into text files, and exported to PDF for Atlas.ti software version 22.0, where data organization and coding were performed.

### Data Analysis

Data were analyzed using the thematic-categorical content analysis technique^(15)^, which includes the phases of pre-analysis, material exploration, and treatment of results, inference, and interpretation. In the first phase, the research team conducted a preliminary reading of the transcriptions, and the study corpus was established based on criteria of exhaustiveness, representativeness, homogeneity, pertinence, and exclusivity. The transcriptions were individually inserted into Atlas.ti version 22.0 software for the selection and organization of textual excerpts (quotations).

During the material exploration phase, exhaustive readings of the content were conducted, and thematic coding was performed in the software based on the registration units (words, phrases, or paragraphs related to the content and context: repercussions of the COVID-19 pandemic on the development of TB detection and control actions in PHC). Themes were not pre-established but derived from the data through the identification of core meanings, to which codes were assigned. The coding was conducted independently by two researchers and later discussed by the team. In the final stage, the codes were grouped by similarity into code groups and, finally, into thematic categories.

For data interpretation, the theoretical framework of vulnerability^(16)^ was used, specifically the programmatic dimension, which refers to institutional components and how they act to mitigate or amplify individual and social vulnerability conditions. It encompasses the realization (or lack thereof) of the commitment of governments, programs, and services, the resources allocated, the values and competencies of professionals, the monitoring and evaluation of health actions, and the conditions for their maintenance.

## RESULTS

Eleven health professionals participated in the research, and their sociodemographic characteristics are summarized in Chart 1.

**Chart 1:** Sociodemographic characteristics of participants, Florianópolis, Santa Catarina, Brazil, 2022-2023

Characteristics	Results	
Age	Average (in years)	Minimum - Maximum (in years)
	41.1	30 - 50
Sex	Female	Male
	10 (90.9%)	01 (9.1%)
Professional category	Nurse	Doctor
	09 (81.8%)	02 (18.2%)
Length of experience in PHC	1 to 5 years	More than 5 years
	01 (9.1%)	10 (90.9%)
Length of service at the unit	2 to 5 years	More than 5 years
	06 (54.5%)	05 (45.5%)

*PHC - Primary Health Care.*

Chart 2 presents the 26 codes generated in Atlas.ti, along with their frequencies of occurrence, code groups, and the two major thematic categories that emerged from the data.

**Chart 2:** Codes (with respective frequencies of occurrence), code groups, and thematic categories

Codes (frequency of occurrence)	Code Groups	Categories
Health professionals’ illness (7)	Health professionals’ conditions	Repercussions of the pandemic on the work process and health conditions of professionals
Health professionals’ medical certificates/leave (7)		
Health professionals’ overload (5)		
Insufficient material resources/PPE (1)		
Sudden increase in work demand (6)	Changes in the work process/routine of the unit caused by the COVID-19 pandemic	
Impairments in carrying out APS-related actions and monitoring other diseases (beyond TB) (5)		
Provision of remote consultations (4)		
Insufficient human resources (4)		
Need for service adaptations to attend respiratory/COVID-19 symptomatic patients (4)		
Need for changes in the work process (3)		
Absence of clinical protocols for COVID-19 at the beginning (2)		
Interruption of team meetings (1)		
Internal organization and previous protocols as facilitators of work process reorganization (1)		
Implementation of telework (1)		
Interruption of home visits (5)	Direct repercussions of the pandemic on tuberculosis control actions	Invisibility of tuberculosis in PHC in a pandemic scenario
Interruption of DOT (4)		
Clinical focus solely on COVID-19 (4)		
Decreased user demand (related to TB) (3)		
Impairments in users’ access to health services (3)		
Maintenance of TB treatment, with adaptations (3)		
Differential diagnosis between tuberculosis and COVID-19 (2)		
Decreased availability of diagnostic tests for TB (1)		
Late detection and notification of TB (1)		
Underreporting of TB cases (1)		
Interruption of sputum collection in the unit (1)		
Treatment supervision through telemonitoring (1)		

*PHC - Primary Health Care; DOT - Directly Observed Treatment; TB - Tuberculosis.*

### Repercussions of the pandemic on the work process and health conditions of professionals

This category presents the difficulties and challenges pointed out by the participants concerning the work process in PHC during the pandemic. The sudden and significant increase in work demand and the insufficiency of human, material, and structural resources are highlighted:

*I think the demand for appointments increased a lot* [...] *the demand became so great that at a certain point we created a work schedule exclusively for COVID. We closed part of the unit so that the care could be exclusive.* (E10)

*It was very difficult, very difficult! The lack of staff and the demand increased threefold; I couldn’t monitor other diseases, and it was very hard.* (E3)

Changes in the physical space, the high demand for care, the virulence of COVID-19, combined with the significant overload due to both the increased demand and the deficits in the workforce, contributed significantly to the physical and mental illness of professionals.

*There were many people, a lot of patient overload. Everyone was emotionally affected; we had many colleagues on leave due to chronic illnesses* [...] *I had COVID twice.* (E8)

*So, we experienced all possible feelings from the beginning of the pandemic. Feelings of helplessness, fear. Many professionals who did not take controlled medication started using these medications because they were traumatized by everything they saw. I think everyone who worked on the front lines like I did has had their mental health affected in some way. Many professionals who were at risk had to go to telework, so they stopped being physically present at the unit to work, I think about 30 to 40% of the team had to go home because they had some comorbidity* [...] *we went through a period of a lot of exhaustion, stress, burnout.* (E9)

*It was a very high demand; we didn’t have clinical security yet, there was a lot of uncertainty at the beginning, before the main protocols started to appear.* (E10)

The fear of the unknown, of illness and death due to COVID-19, and the feeling of helplessness were highlighted by the professionals as difficult and stressful emotions they experienced. Clinical insecurity, especially at the beginning of the pandemic when the demand for care was increasing and the protocols and care flows were still being developed and implemented, was also emphasized.

The high level of stress was related by the participants to the insufficient physical infrastructure and resources to provide adequate and safe care, as well as to the structural changes and care flows that were abruptly established to attend to respiratory symptomatic patients suspected of having COVID-19:

*It was very stressful because our unit is far from centers, hospitals, and the emergency room. We had to adapt our entire unit, the respiratory symptomatic ward with oxygen and masks. During the peak of the pandemic, we started receiving people with dyspnea, respiratory failure, and we had to monitor them here at the unit with little structure for that. Sometimes we stayed until eight, nine o’clock at night trying to transfer the patient to the hospital and there was no ambulance.* (E5)

*It was challenging. Challenging because we had to change the flow of appointments a bit, and everything was very new.* [...] *it ended up changing the physical structure of the unit so that we could attend to these respiratory symptomatic patients.* (E7)

The scarcity and rationing of personal protective equipment (PPE) were also difficulties experienced by the workers, who had to deal with changes in recommendations regarding the use and durability of materials and the use of homemade materials without quality control. The shortage of PPE, combined with the lack of a defined protocol for attending to patients at the beginning of the pandemic, increased the risk of illness among professionals.

*In the beginning, it was very difficult because we didn’t have a defined protocol to attend to the patients and there was a significant lack of PPE. We even had to sign for N95 masks and use the same mask for 15 days. We didn’t have Face Shields available at first, and we received some poorly made ones from some institutions, but it was what we had, and we used them.* (E9)

In the work process, actions aimed at health promotion and disease prevention, which are some of the pillars of PHC, were significantly impacted:

[...] *it was very complicated because we stopped doing what we do, which is primary health care. We stopped making home visits, team meetings had to stop, routine care* [...] *we had to postpone the management of non-communicable chronic diseases a bit.* (E8)

Among the strategies adopted by the health teams in an attempt to mitigate the damage caused by the pandemic, the use of technological resources such as telemedicine, virtual consultations, and communication with patients via WhatsApp stood out. These strategies enabled professionals to conduct virtual appointments/follow-ups and maintain a connection with the users during the period of social distancing.

*The main change that came with this pandemic was digital access, which we did not have before. Each health team has a phone with WhatsApp, which is our main tool for virtual care. Patients contact us for any type of demand, whether to schedule an in-person consultation, a video call, or to send test results, request prescription renewals* [...]*, so it became a routine for accessing all services.* (E10)

In this context, the prominent role of nurses in PHC stands out, as evidenced by their leadership in the complex process of reorganizing the unit, a crucial aspect for ensuring access and assistance to users during the pandemic:

*The internal organization facilitated the work process and helped a lot. Florianópolis is already known for the collaborative work of nurses, as we have protocols that support what nurses can do. The increased demand caused a lot of stress due to work pressure, but the organization made it smoother.* (E1)

### Invisibility of Tuberculosis in Primary Health Care during the pandemic

This category presents the changes and/or interruptions in the provision of TB control actions in PHC during the pandemic. Health services continued to use the existing care protocol in the municipality (prior to the pandemic). However, several participants reported that there was possibly negligence in diagnosing other diseases in the pandemic context, considering that the clinical focus of the professionals was heavily directed toward COVID-19. This may have led to a process of invisibility of TB in this scenario.

*When we had symptoms that could suggest a tuberculosis diagnosis, we were very focused on COVID-19. So, perhaps TB was somewhat neglected by the vast majority of professionals.* (E4)

[...] *we were thinking a lot about COVID, which hindered the detection of cases of all other diseases because when we focus a lot on one thing, it’s the only thing we see, and we end up being somewhat limited to thinking only about that.* (E8)

Directly observed treatment (DOT) was interrupted, as most treatment control appointments were conducted via telehealth. Medications continued to be dispensed at the health unit, but without direct and daily usage monitoring. One of the participants reported conducting weekly control by observing the medication blister pack (counting the number of pills taken).

*As we greatly restricted in-person appointments, most were online, and DOT was compromised because we couldn’t do it at the health unit.* (E9)

[...] *sometimes, we opted to deliver more medication and do a weekly observation of the intake, for example. The form of control we used was that the patient brought the empty blister packs, without the pills, so we could have at least a minimal sense of medication control.* (E10)

There were disruptions in the routine of active case finding for respiratory symptomatic (RS) individuals for TB, as home visits by community health agents were reduced or suspended. Additionally, when a service user visited the unit with flu-like symptoms and reported coughing for more than three weeks, although a sputum test was requested, the collection of the first sample was not performed at the health unit as recommended. In this context, the management of test requests and their execution was impaired.

*We kept requesting* [the sputum test]*. What we lost a lot was control over how many people were actually going to be tested and especially active case finding because the health agents were not doing home visits.* (E2)

*If there was a suspicion of tuberculosis, we would request sputum and an X-ray during the consultation, but we chose not to collect the sample at the time of the consultation and asked the patient to do it at home* [...] *to avoid exposure.* (E10)

It is noteworthy that some actions had not yet been fully resumed in the first quarter of 2023, such as sputum collection at the unit, the workflow of the laboratory that conducts TB tests, active case finding, and DOT. These actions directly impact the detection, treatment outcomes, and therefore the epidemiology of the disease in the municipality.

*The laboratory is still there, serving as the municipal reference for COVID PCR collection, and we haven’t regained that physical space for TB. We stopped doing the collection at the unit. Now we ask the patient to collect the sample at home.* (E10)

## DISCUSSION

The data show that the pandemic scenario caused changes in the work processes of the units, necessitating abrupt adaptations. It also caused physical and psychological harm to professionals and disruptions in disease control actions, which were reduced or discontinued.

Most of the key informants were nurses, reaffirming the relevance of this profession in TB control actions in PHC. Nursing plays a leading role in the prevention, detection, and treatment of the disease through comprehensive interventions aimed at different spheres: individual, family, and social. Nursing participates in the design, implementation, and evaluation of public policies, direct assistance to people with TB, health education for individuals and the community, among other actions^(17)^.

The International Council of Nurses (ICN) published the report “Recover to Rebuild: Investing in the Nursing Workforce for Health System Effectiveness” in 2023^(18)^, presenting concerning evidence about the impact of the COVID-19 pandemic on the nursing workforce, such as increasing rates of nurses reporting burnout and leaving the profession. Based on the results of more than 100 studies, including systematic reviews, the report indicated that 40% to 80% of nurses experienced symptoms of psychological distress during the pandemic.

Among these studies, a global survey conducted in 2022 revealed that 20% to 38% of responding nurses in the USA, the UK, Singapore, Japan, France, Brazil, and Australia expressed their intention to leave their current roles in direct patient care within the next year. Stress, burnout, and absenteeism were highlighted as symptoms of the current work and health conditions of nurses. The report discusses the vital yet dangerous role nurses played during the pandemic and emphasizes that without sufficient investment to support these professionals, there will be no effective recovery and rebuilding of health systems^(18)^.

The reality described by the participants in this study is similar to that found in other studies^(19,20)^, which also highlighted the unhealthy conditions of professional practice during the pandemic, emphasizing the lack of adequate and/or sufficiently available PPE, lack of basic supplies, insufficient number of professionals, and long working hours under significant psychological stress, and even physical and mental illness.

Human resources are one of the main pillars for achieving the goals of TB elimination. The importance of nursing professionals in developing disease control actions brings to light the imminent need for the recovery and appreciation of the nursing workforce, which requires urgent institutional and governmental strategies and actions, with the formulation and implementation of public policies for the prevention, containment, diagnosis, and treatment of occupational illness^(17)^.

The findings of this investigation demonstrated that the main TB control actions were altered due to the reassignment of human resources to frontline COVID-19 efforts, social distancing, and changes in the organization of workflow. These data are corroborated by other studies^(21-23)^, which also identified declines in the performance of disease control actions compared to pre-COVID-19 performance in various locations in Brazil and around the world.

The reduction in case detection globally could lead to an increase in mortality from the disease, particularly affecting the most vulnerable people^(21)^. In Brazil, a reduction in confirmed and reported cases of pulmonary TB was observed in all regions except the North during the pandemic period^(24)^. These data corroborate the study by the Global Tuberculosis Network, which indicated that the TB diagnosis rate and latent infection decreased during the COVID-19 pandemic in many countries^(25)^.

Active case finding of RS, which was interrupted in the studied scenario, is an action that effectively contributes to the detection of TB cases and the rapid initiation of treatment, aiming to break the chain of transmission and reduce the incidence of the disease in the long term. Intensifying active case finding, considering the particularities of the most vulnerable populations in the territories, is one of the main strategies of the National Plan for Ending Tuberculosis^(26)^.

Adherence to treatment is another relevant aspect for the prevention and control of the disease, as low adherence can lead to the development of drug-resistant TB and favor the continuation of the transmission chain^(27)^. One of the strategies recommended by the WHO and the Ministry of Health to improve adherence is the adoption of DOT^(26)^.

Studies^(28-30)^ point to the benefits and efficiency of DOT in TB control. One study conducted in Curitiba-PR showed that DOT played a fundamental role in controlling the disease, with a reduction in the number of new cases, a decrease in the treatment interruption rate, and a reduction in TB-related deaths^(29)^. Such evidence highlights the relevance of resuming and reorganizing this strategy to combat the disease in the post-pandemic period.

The health care protocols in Florianópolis were cited as a strong point, providing important support for the internal organization of the service at the beginning of the pandemic. Despite this, it was identified that the municipal TB care protocol was not sufficient to maintain the quantity and quality of actions during the pandemic context.

In March 2020, the General Coordination of Respiratory Disease Surveillance recommended the organization of the local health care network to ensure that people with signs and symptoms of TB had access to health services and laboratory tests. It also recommended the organization of the health network and guidance on diagnosis, reinforcing the importance of bilateral TB-COVID screening, in addition to the use of locally available technological strategies to contact the user^(31)^.

However, there were difficulties in implementing these recommendations, mainly due to insufficient human and material resources, as well as the intense shift in focus of health professionals and health agencies towards COVID-19. This led to a situation where TB became largely invisible, especially regarding detection actions.

The recommendation most effectively implemented in the studied scenario was the use of technological strategies for monitoring service users. These strategies were also successfully used in other parts of Brazil and the world^(32)^. The experiences point to some interesting paths for the future use of technology and telecommunication in health care, reinforcing the need for greater investment in science and technology and in strategies to address access inequalities^(33)^.

The damages caused by the pandemic highlight the need for institutional and governmental programmatic commitment to comprehensive and longitudinal care, with continued access to diagnosis and treatment, as well as surveillance of health issues in territories, especially those where the most vulnerable populations live and work. PHC has a strategic role in combating TB through active case finding of RS, early diagnosis, timely treatment, and monitoring through DOT, aiming to increase cure rates and reduce the transmission chain and the emergence of multidrug-resistant TB^(34)^.

Strengthening PHC is indispensable for advancing disease control, as ineffective follow-up by PHC teams can delay diagnosis and favor treatment interruption. Professionals must adopt a proactive, welcoming, and resolute attitude in caring for TB patients and their families, considering each encounter as an opportunity to create or continue a collaborative therapeutic project, where the professional and the service share responsibility with the user^(35)^.

Thus, it is necessary and urgent to reorganize the TB care network, resume suspended and/or hindered actions, value the health professionals involved in these actions, and strengthen prevention, diagnosis, treatment, and surveillance strategies, as well as integrated intersectoral actions, to overcome the pandemic’s impacts and fulfill the commitments made in the End TB Strategy and the SDGs.

### Study limitations

Data collection was conducted at the end of the pandemic period (December 2022 to April 2023), so some aspects of the professional experience during the pandemic may have been forgotten or reinterpreted by the participants. Another limitation is the absence of feedback from the interview transcripts to the participants for comments and/or corrections. Finally, it should be noted that the data come from a single scenario, and the results may not be generalizable to all populations or contexts.

### Contributions to the Health Field and Nursing

The study contributes to understanding the challenges faced by PHC professionals in offering and carrying out TB control actions during the pandemic and points out relevant aspects that should be prioritized in the resumption and reorganization of these actions. Health policies should consider the pandemic’s repercussions, start from the current reality experienced by the actors (health professionals) directly involved in control actions, and incorporate scientific evidence to restructure coping strategies.

The results of this research can provide subsidies for the development of policies and strategies aimed at resuming and reorganizing TB control and elimination actions as a public health problem, mainly at the municipal and state levels, as well as policies aimed at strengthening and valuing PHC and the health workforce, with a particular emphasis on nursing.

## FINAL CONSIDERATIONS

The findings evidenced programmatic vulnerability, manifested in the unpreparedness and lack of resources of various governmental levels and health services to handle the pandemic as a public health emergency without severely compromising the provision of essential services, such as TB control actions. Additionally, there was a lack of monitoring and care actions for the health workforce, especially nursing professionals.

The ambitious goals of the SDGs and the End TB strategy require the implementation of actions and policies focused on the comprehensiveness and intersectorality of care, the availability of material and human resources allocated strategically and effectively, the appreciation and recovery of the health workforce that carries out disease control actions, and the intensification of investment in research and innovation so that favorable outcomes and the achievement of goals are possible. This is the path forward; we must follow it.
