# Supplementary material for: Repercussions of the pandemic on tuberculosis control actions from the perspective of health professionals
Source: Rev Bras Enferm. 2024 Dec 13;77(5):e20230477. doi: 10.1590/0034-7167-2023-0477 (PMC11654224; doi:10.1590/0034-7167-2023-0477)
Supplement: Translation

# Translation: Repercussões da pandemia nas ações de controle da tuberculose na perspectiva de profissionais da saúde

**DOI:** 10.1590/0034-7167-2023-0477pt

**Authors:** Sabrina da Silva de Souza, Roxana Isabel Cardozo Gonzales, Hellen Cristina Sthal, Daiane Cardoso da Silva, Marcela Flavia Lopes Barbosa, Tatiana Conceição

**Affiliations:** I Universidade Federal de Santa Catarina. Florianópolis, Santa Catarina, Brasil; II Universidade Federal de Goiás. Goiânia, Goiás, Brasil

**Keywords:** Tuberculose, Atenção Primária à, Saúde, COVID-19, Avaliação em Saúde, Recursos Humanos

## Abstract

**Objetivos:**

analisar as repercussões da pandemia da COVID-19 nas ações de controle da tuberculose, na perspectiva de profissionais de saúde da Atenção Primária à Saúde.

**Métodos:**

estudo descritivo, com abordagem qualitativa, realizado no período de novembro de 2022 a abril de 2023, por meio de entrevistas semidirigidas, com 11 profissionais informantes-chave de unidades básicas de saúde de uma capital brasileira. Os dados foram organizados no *software* Atlas.ti 22.0 e submetidos à análise de conteúdo temático-categorial.

**Resultados:**

o cenário pandêmico provocou alterações no processo de trabalho, com necessidade de adaptações abruptas, e causou prejuízos na saúde dos profissionais e nas ações de controle da tuberculose, que foram reduzidas ou descontinuadas.

**Considerações Finais:**

evidenciou-se despreparo e falta de recursos das diversas instâncias governamentais e dos serviços de saúde para lidar com a situação de emergência em saúde pública, sem que houvesse graves prejuízos na oferta dos serviços essenciais.

## INTRODUÇÃO

A tuberculose (TB) é uma doença altamente contagiosa, que permanece como um grave problema de saúde pública global, especialmente nos países em desenvolvimento. Em 2022, 10,6 milhões de pessoas adoeceram por TB no mundo e cerca de 1,3 milhão morreram pela doença^(1)^. Na região das Américas, o Brasil concentra a maior carga de TB (33%)^(2)^ e integra a lista de países prioritários da Organização Mundial da Saúde (OMS) para o período 2021-2025, por apresentar alta carga de TB e alta carga de coinfecção TB/HIV^(1)^. Em 2022, aproximadamente 78 mil pessoas adoeceram por TB no Brasil e cerca de 5 mil morreram pela doença^(3)^.

Devido à magnitude epidemiológica, econômica e social da TB, sua eliminação como problema de saúde pública está prevista nos Objetivos de Desenvolvimento Sustentável (ODS), estabelecidos pela Organização das Nações Unidas (ONU) em 2015. No mesmo ano, a OMS lançou a estratégia global *End TB*, que visa à redução de 90% da incidência e 95% da taxa de mortalidade pela doença até 2035^(4)^. Em 2017, o Brasil publicou o Plano Nacional pelo Fim da Tuberculose como Problema de Saúde Pública^(5)^. Contudo, as metas da primeira fase do plano (2017-2020) não foram alcançadas, e a crise sanitária e social agravada pela pandemia da COVID-19 tornou ainda mais complexo e distante o alcance das metas das próximas fases^(2)^.

Em 2020, ano de início da pandemia, estima-se que houve uma redução de 18% na detecção de casos de TB globalmente, em comparação com 2019^(3,6)^. No Brasil, houve uma redução de 12,1% no coeficiente de incidência da doença, que passou de 37,9 casos/100 mil hab., em 2019, para 33,3, em 2020. Em 2021, foram registrados 34,9 casos/100 mil hab. e, em 2022, 36,3, apontando uma recuperação parcial na detecção, contudo com valores ainda inferiores aos observados nos anos pré-pandêmicos^(3)^.

Em 2019, a taxa de interrupção do tratamento da TB foi de 12%, aumentando para 12,7% em 2020 e para 14,6% em 2021^(3,7,8)^. Houve um aumento de 0,8% no número de óbitos entre 2019 e 2020. Em 2021, o aumento foi de 12,0% em relação a 2019^(3)^. Percebe-se que, com o avanço da COVID-19 nos anos de 2020 e 2021, houve piora expressiva nos indicadores da TB.

Os serviços de saúde brasileiros vivenciaram um aumento explosivo em sua demanda, extrapolando a capacidade estrutural, inclusive na Atenção Primária à Saúde (APS), que desempenha um papel fundamental nas ações de prevenção, vigilância, detecção e tratamento da TB. A redução da quantidade e qualidade de suas ações de controle resulta em diagnósticos tardios, tratamentos inadequados e aumento da disseminação nos territórios, o que pode gerar altos custos nos sistemas de saúde e, especialmente, na vida das pessoas e famílias acometidas^(6)^, além do agravamento do perfil epidemiológico do país^(2)^.

Apesar da relevância da APS para o controle da TB e da magnitude dos impactos gerados pela pandemia, ainda são escassas as publicações científicas sobre o tema, principalmente na perspectiva dos atores sociais (profissionais de saúde) que atuaram na APS nesse cenário. Assim, é fundamental dar visibilidade a esses profissionais de saúde e aos desafios enfrentados por eles no período pandêmico, refletindo sobre o processo de trabalho na APS, a atenção e cuidado às pessoas com TB e a relevância da força de trabalho em saúde para a reorganização das ações de controle da doença no pós-pandemia.

## OBJETIVOS

Analisar as repercussões da pandemia da COVID-19 nas ações de controle da tuberculose, na perspectiva de profissionais de saúde da Atenção Primária à Saúde.

## MÉTODOS

### Aspectos éticos

A pesquisa foi aprovada pelo Comitê de Ética em Pesquisa (CEP) do Hospital Escola da Universidade Federal de Goiás, instituição proponente do projeto Matriz. Para garantir o sigilo da identidade dos participantes, estes foram identificados pela letra “E” seguida da ordem numérica (de E1 a E11). Todos foram esclarecidos sobre os objetivos e procedimentos da pesquisa e só foram entrevistados após leitura e assinatura do Termo de Consentimento Livre e Esclarecido (TCLE).

### Tipo de estudo

Estudo descritivo, com abordagem qualitativa^(9)^, inserido na pesquisa multicêntrica de método misto intitulada “Repercussão da pandemia da COVID-19 na tuberculose em capitais brasileiras: realidade e novas perspectivas na Atenção Primária”, que está sendo desenvolvida em seis capitais brasileiras (Florianópolis, Goiânia, João Pessoa, Maceió, Porto Velho e São Paulo), financiada pelo Conselho Nacional de Desenvolvimento Científico e Tecnológico (CNPq) - Chamada nº 18/2021. Foram adotadas as recomendações do *Consolidated Criteria for Reporting Qualitative Research* (COREQ)^(10)^.

### Cenário do estudo

O estudo foi realizado nas unidades de APS de Florianópolis, capital do Estado de Santa Catarina, na região Sul do Brasil. O município conta com 51 unidades básicas de saúde, divididas em quatro distritos: continente, centro, norte e sul^(11)^ e atingiu, em 2020, 76,75% de cobertura da atenção básica^(12)^. Em 2022, apresentou coeficiente de incidência de TB de 33 casos novos/100 mil hab.^(3)^. A atenção a essa doença no município é descentralizada, sendo realizada em todas as unidades de saúde.

### Fonte de dados

Participaram os profissionais de saúde indicados como informantes-chave (pelo gestor ou pela equipe) das unidades de APS selecionadas por amostragem aleatória simples, a partir da amostra de unidades que compuseram a etapa quantitativa da pesquisa matriz. Eles foram convidados por telefone, correio eletrônico ou *WhatsApp*.

Considerou-se como critério de inclusão estar ativo em serviço de APS por no mínimo três meses no período pandêmico. Foram excluídos os profissionais afastados de suas atividades por qualquer motivo no período da coleta de dados. A amostra^(13)^, composta por 11 informantes-chave, foi definida por meio da técnica de saturação de dados^(14)^. A saturação foi avaliada e discutida por duas pesquisadoras da equipe. Não houve recusas ou desistências de participação.

### Coleta e organização dos dados

A coleta foi realizada no período de novembro de 2022 a abril de 2023, por meio de entrevistas semidirigidas^(13)^, baseadas em um roteiro elaborado pelos pesquisadores, contendo dados de identificação (como sexo, idade), dados profissionais (profissão e tempo de atuação na APS e na unidade), além de questões abertas sobre a experiência da atuação profissional no contexto da COVID-19; sobre a oferta de ações de controle da TB e sobre as repercussões da pandemia nessas ações. O roteiro foi revisado por dois especialistas e aplicado a três profissionais enfermeiros, que não compuseram a amostra, para avaliar a clareza das questões norteadoras da entrevista.

As entrevistas foram realizadas em sala reservada, nas dependências das unidades de saúde, mediante agendamento prévio, de acordo com a preferência e disponibilidade de dia e horário do profissional de saúde. Cada participante foi entrevistado uma única vez. Todas as entrevistas foram conduzidas por pesquisadoras enfermeiras integrantes da equipe (docentes e discentes de pós-graduação), devidamente treinadas. Não foi estabelecido relacionamento prévio com os participantes antes do início da pesquisa. As pesquisadoras e os objetivos e procedimentos da pesquisa foram apresentados aos participantes no momento do convite para participação.

Utilizou-se diário de campo para registrar as ocorrências e as observações das pesquisadoras. As notas de campo foram registradas imediatamente após a realização de cada entrevista. As entrevistas tiveram duração média de 10 minutos, foram gravadas, com aparelho gravador de voz, transcritas na íntegra pelas pesquisadoras em arquivos de texto e exportadas em PDF para o *software* Atlas.ti versão 22.0, no qual foi realizada a organização e a codificação dos dados.

### Análise dos dados

Os dados foram analisados com base na técnica de análise de conteúdo temático-categorial^(15)^, que contempla a fase de pré-análise, exploração do material e tratamento dos resultados, inferência e interpretação. Na primeira fase, realizou-se a leitura flutuante das transcrições, pela equipe de pesquisa, e constituiu-se o *corpus* do estudo, tendo como critérios: exaustividade, representatividade, homogeneidade, pertinência e exclusividade. As transcrições foram inseridas, individualmente, no *software* Atlas.ti versão 22.0. para seleção e organização dos recortes textuais *(quotations)*.

Na fase de exploração do material, foram realizadas leituras exaustivas do conteúdo e a codificação temática no *software*, a partir das unidades de registro (palavras, frases ou parágrafos relacionados ao conteúdo e contexto: repercussão da pandemia COVID-19 no desenvolvimento das ações de detecção e controle da TB na APS). Os temas não foram estabelecidos antecipadamente, foram derivados dos dados, a partir da identificação dos núcleos de sentido, aos quais foram atribuídos códigos. A codificação foi realizada, de maneira independente, por duas pesquisadoras e posteriormente discutida pela equipe. Na última etapa, os códigos foram agrupados, por similitude, em grupos de códigos e, por fim, em categorias temáticas.

Para interpretação dos dados, utilizou-se o referencial teórico da vulnerabilidade^(16)^, especificamente a dimensão programática, que se refere aos componentes institucionais e como eles atuam para amenizar ou ampliar as condições de vulnerabilidade individuais e sociais. Engloba a concretização (ou não) do compromisso dos governos, programas e serviços, os recursos destinados, os valores e as competências dos profissionais, a forma como ocorre o monitoramento e a avaliação das ações de saúde, além das condições para a sua manutenção.

## RESULTADOS

Participaram da pesquisa 11 profissionais de saúde, cujas características sociodemográficas estão sintetizadas no Quadro 1.

**Quadro 1:** Características sociodemográficas dos participantes, Florianópolis, Santa Catarina, Brasil, 2022-2023

Características	Resultados	
Idade	Média (em anos)	Mínima - Máxima (em anos)
	41,1	30 - 50
Sexo	Feminino	Masculino
	10 (90,9%)	01 (9,1%)
Categoria profissional	Enfermeiro(a)	Médico (a)
	09 (81,8%)	02 (18,2%)
Tempo de experiência na APS	De 1 a 5 anos	Maior que 5 anos
	01 (9,1%)	10 (90,9%)
Tempo de atuação na unidade	De 2 a 5 anos	Maior que 5 anos
	06 (54,5%)	05 (45,5%)

*APS - Atenção Primária à Saúde.*

O Quadro 2 apresenta os 26 códigos gerados no *Atlas.ti*, com suas frequências de ocorrência, os grupos de códigos e as duas grandes categorias temáticas que emergiram dos dados.

**Quadro 2:** Códigos (com as respectivas frequências de ocorrência), grupos de códigos e categorias temáticas

Códigos (frequência de ocorrência)	Grupos de códigos	Categorias
Adoecimento dos profissionais de saúde (7)	Condições de saúde dos profissionais	Repercussões da pandemia no processo de trabalho e nas condições de saúde dos profissionais
Atestados/afastamentos de profissionais de saúde (7)		
Sobrecarga dos profissionais de saúde (5)		
Recursos materiais/EPIs insuficientes (1)		
Aumento repentino da demanda de trabalho (6)	Alterações no processo de trabalho/rotina da unidade ocasionadas pela pandemia da COVID-19	
Prejuízos na realização das ações inerentes à APS e acompanhamento de outras doenças (para além da TB) (5)		
Realização de atendimentos remotos (4)		
Insuficiência de recursos humanos (4)		
Necessidade de adaptações do serviço para atender os sintomáticos respiratórios/COVID-19 (4)		
Necessidade de alterações no processo de trabalho (3)		
Ausência de protocolos clínicos para a COVID-19 no início (2)		
Interrupção da realização das reuniões de equipe (1)		
Organização interna e protocolos prévios como facilitadores da reorganização do processo de trabalho (1)		
Realização de teletrabalho (1)		
Interrupção da realização das visitas domiciliares (5)	Repercussões diretas da pandemia nas ações de controle da tuberculose	Invisibilidade da tuberculose na APS em cenário pandêmico
Interrupção do TDO (4)		
Olhar clínico focado apenas na COVID-19 (4)		
Diminuição da procura dos usuários (referente à TB) (3)		
Prejuízos no acesso dos usuários ao serviço de saúde (3)		
Manutenção do tratamento da TB, com adaptações (3)		
Diagnóstico diferencial entre tuberculose e COVID-19 (2)		
Diminuição da oferta de exames diagnósticos para TB (1)		
Detecção e notificação tardia da TB (1)		
Subnotificação dos casos de TB (1)		
Interrupção da coleta de escarro na unidade (1)		
Supervisão do tratamento por telemonitoramento (1)		

*APS - Atenção Primária à Saúde; TDO - Tratamento diretamente observado; TB - Tuberculose.*

### Repercussões da pandemia no processo de trabalho e nas condições de saúde dos profissionais

Esta categoria apresenta as dificuldades e desafios apontados pelos participantes, com relação ao processo de trabalho na APS durante a pandemia. Destaca-se o aumento repentino e expressivo da demanda de trabalho e a insuficiência de recursos humanos, materiais e estruturais:

*Eu acho que aumentou muito a demanda de atendimentos* [...] *a demanda chegou a ser tão grande que em determinado momento criamos uma escala de trabalho exclusiva para a COVID. Fechamos uma parte da unidade para o atendimento ficar exclusivo.* (E10)

*Foi bem difícil, muito difícil! A falta de funcionários e a demanda aumentou três vezes mais; não consegui fazer vigilância das outras doenças e foi bem difícil.* (E3)

As mudanças no espaço físico, a alta demanda de atendimentos, a virulência da COVID-19, somadas à grande sobrecarga, tanto pelo aumento da demanda quanto pelos déficits na força de trabalho, contribuíram significativamente para o adoecimento físico e psíquico dos profissionais.

*Foi muita gente, muita sobrecarga de pacientes. Todo mundo ficou ruim emocionalmente, tivemos muitos colegas afastados por doenças crônicas* [...] *eu tive duas vezes COVID.* (E8)

*Assim, tivemos todos os sentimentos possíveis do início da pandemia. Sentimento de impotência, medo. Muitos profissionais que não tomavam medicamento controlado, começaram a fazer uso dessas medicações a partir da pandemia, por terem ficado traumatizados com tudo que viram. Acho que todos que trabalharam na linha de frente como eu, de alguma forma têm uma saúde mental afetada. Muitos profissionais que eram do grupo de risco precisaram ir para o teletrabalho, então deixaram de estar presencialmente na unidade para trabalhar, acho que por volta de 30, 40% da equipe teve que ir pra casa porque tinha alguma comorbidade* [...] *a gente passou por um momento de muito desgaste, estresse, burnout.* (E9)

*Era uma demanda muito grande, a gente ainda não tinha segurança da clínica, tinha muita insegurança no começo, antes dos principais protocolos começarem a aparecer.* (E10)

O medo do desconhecido, do adoecimento e da morte por COVID-19, e a sensação de impotência, foram destacados pelos profissionais como sentimentos difíceis e estressantes vivenciados. A insegurança clínica, principalmente no início da pandemia, quando a demanda por atendimento era crescente e os protocolos e fluxos de atendimento ainda estavam em fase de elaboração e implementação, também foi ressaltada.

O nível elevado de estresse foi relacionado, pelos participantes, à insuficiência de estrutura física e de recursos para oferecer uma assistência adequada e livre de danos, bem como às mudanças estruturais e dos fluxos de atendimento, que foram estabelecidas abruptamente para atender os sintomáticos respiratórios com suspeita de COVID-19:

*Foi bem estressante, porque a nossa unidade é longe de centros, hospitais e do pronto socorro. Tivemos que adaptar toda nossa unidade, ala de sintomático respiratório com oxigênio, máscaras. E no forte da pandemia nós começamos a receber pessoas com quadro de dispneia, insuficiência respiratória e tivemos que monitorar aqui na unidade, com pouca estrutura pra isso. Algumas vezes ficamos até oito, nove horas da noite para tentar transferir o paciente para o hospital e não tinha ambulância.* (E5)

*Foi desafiador. Desafiador porque tivemos que mudar um pouco os fluxos de atendimentos e era tudo muito novo.* [...] *acabou mudando a estrutura física da unidade, para que pudéssemos atender esses pacientes sintomáticos respiratórios.* (E7)

A escassez e racionamento de equipamentos de proteção individual (EPIs) também foi uma dificuldade experienciada pelos trabalhadores, que precisaram lidar com alterações referentes às recomendações de uso e durabilidade dos materiais e com a utilização de materiais de produção artesanal, sem controle de qualidade. A escassez de EPIs, somada à falta de um protocolo definido para atender os pacientes no início da pandemia, aumentou o risco de adoecimento dos profissionais.

*No começo foi bem difícil porque a gente não tinha um protocolo definido para atender os pacientes e faltava muito EPI. Até N95 a gente tinha que assinar e ficar com a máscara durante 15 dias. Face Shield não tivemos disponível no começo e nós ganhamos algumas bem malfeitas de algumas instituições, mas era o que tínhamos e nós usávamos.* (E9)

No processo de trabalho, as ações voltadas para promoção da saúde e prevenção de agravos, que são alguns dos pilares da APS, foram bastante prejudicadas:

[...] *bem complicado porque a gente parou de fazer o que a gente faz, que é atenção primária em saúde. Deixamos de fazer visita domiciliar, reuniões de equipe tiveram que parar, atendimento assim de rotina* [...] *dos casos de doenças crônicas não transmissíveis tivemos que postergar um pouco.* (E8)

Dentre as estratégias adotadas pelas equipes de saúde, na tentativa de mitigar os prejuízos causados pela pandemia, destacou-se a utilização de recursos tecnológicos, como a telemedicina, atendimentos virtuais e comunicação com os pacientes por *WhatsApp*. Tais estratégias possibilitaram aos profissionais a realização de atendimentos/acompanhamentos virtuais e a manutenção do vínculo com o usuário durante o período de distanciamento social.

*A principal mudança que veio com essa pandemia foi o acesso digital, que era uma coisa que não tínhamos antes. Cada equipe de saúde tem um telefone com o WhatsApp, que é a nossa principal ferramenta para esse atendimento virtual, os pacientes entram em contato para qualquer tipo de demanda, seja para agendamento de consulta presencial, por vídeo ou para envio de resultados de exames, solicitação de renovação de receita* [...]*, então virou uma rotina para o acesso de todos os serviços.* (E10)

Nesse contexto, destaca-se o protagonismo do enfermeiro na APS, evidenciado por sua liderança no complexo processo de reorganização da unidade, aspecto que foi crucial para a garantia de acesso e assistência aos usuários durante a pandemia:

*A organização interna facilitou o processo de trabalho, e ajudou muito. Florianópolis já é conhecida pela atuação em conjunto de enfermeiros, pois temos os protocolos que respaldam o que o enfermeiro pode fazer. A demanda aumentou muito o estresse, pela pressão do trabalho, mas a organização fez isso ser mais suave.* (E1)

### Invisibilidade da tuberculose na Atenção Primária à Saúde em cenário pandêmico

Esta categoria apresenta as alterações e/ou interrupções na oferta das ações de controle da TB na APS durante a pandemia. Os serviços de saúde seguiram utilizando o protocolo assistencial vigente no município (anterior à pandemia). Contudo, diversos participantes relataram que possivelmente houve negligência no diagnóstico de outras doenças no contexto pandêmico, considerando que o olhar clínico dos profissionais estava muito direcionado à COVID-19. Isso pode ter levado a um processo de invisibilidade da TB nesse cenário.

*Quando tínhamos sintomas que poderiam despertar para um diagnóstico de tuberculose, estávamos muito enraizados e pensando na questão do COVID-19. Então, talvez tenha ficado um pouco negligenciado por parte da grande maioria dos profissionais.* (E4)

[...] *a gente pensava muito em COVID, atrapalhou a detecção de casos de todas as outras doenças, porque quando a gente pensa muito numa coisa é só aquilo que a gente vê, a gente acaba ficando meio limitado a pensar só naquilo.* (E8)

O tratamento diretamente observado (TDO) foi interrompido, pois a maioria dos atendimentos de controle de tratamento eram realizadas por teleatendimento. Os medicamentos continuaram sendo dispensados na unidade de saúde, porém sem o controle direto e diário da utilização. Um dos participantes relatou ter realizado controle semanal, por meio da observação da cartela do medicamento (contagem do número de comprimidos retirados).

*Como restringimos bastante o atendimento presencial, a maioria era online e o TDO ficou prejudicado porque não conseguíamos fazer na unidade de saúde.* (E9)

[...] *a gente, às vezes, optava por entregar mais medicação e fazer uma observação semanal da tomada, por exemplo. A forma de controle que a gente fazia era que ele trazia as cartelinhas vazias, sem os comprimidos, para a gente ter minimamente uma noção ali, controle da medicação.* (E10)

Houve prejuízos na rotina da busca ativa dos sintomáticos respiratórios (SR) para TB, considerando que as visitas domiciliares, realizadas pelos agentes comunitários de saúde, foram reduzidas ou suspensas. Além disso, quando o usuário do serviço comparecia à unidade com sintomas gripais e relato de tosse há mais de três semanas, embora o exame de escarro fosse solicitado, a coleta da primeira amostra não era realizada na unidade de saúde, como preconizado. Nesse contexto, o controle das solicitações e realização dos exames foi prejudicado.

*Continuamos pedindo* [o exame de escarro]*. O que perdemos muito foi o controle, sobre quantas pessoas, de fato, que iríamos mandar fazer o teste e a busca ativa, principalmente, de busca ativa, porque os agentes de saúde não estavam fazendo visitas domiciliares.* (E2)

*Se houvesse suspeita de tuberculose, pedíamos o escarro, o raio-x, na própria consulta, mas optamos por não fazer a coleta no momento da consulta e pedir para o paciente fazer em casa,* [...] *para evitar a exposição.* (E10)

Destaca-se que algumas ações ainda não haviam sido completamente retomadas, no primeiro trimestre de 2023, como a coleta de escarro na unidade, o fluxo do laboratório que realiza os exames de TB, a busca ativa e o TDO. Tais ações impactam diretamente na detecção, nos desfechos do tratamento e, portanto, na epidemiologia da doença no município.

*O laboratório continua lá, sendo a referência municipal para a coleta do PCR de COVID e não recuperamos esse espaço físico para TB. Paramos de fazer a coleta na unidade. Agora pedimos que o paciente colete em casa.* (E10)

## DISCUSSÃO

Os dados evidenciam que o cenário pandêmico provocou alterações no processo de trabalho das unidades, com necessidade de adaptações abruptas. Causou, ainda, prejuízos à saúde física e psíquica dos profissionais e nas ações de controle da doença, que foram reduzidas ou descontinuadas.

A maioria dos profissionais indicados como informantes-chave foram enfermeiros(as), reafirmando a relevância desse profissional nas ações de controle da TB na APS. A enfermagem desempenha papel de protagonista na prevenção, detecção e tratamento da doença, por meio de intervenções integrais, voltadas às diferentes esferas: individual, familiar e social. A enfermagem participa desde o desenho, implementação e avaliação das políticas públicas, até a assistência direta às pessoas com TB, a educação em saúde dos indivíduos e da coletividade, entre outras ações^(17)^.

O *International Council of Nurses* (ICN) publicou, em 2023, o relatório *Recover to Rebuild: Investing in the Nursing Workforce for Health System Effectiveness* ^(18)^, apresentando evidências preocupantes sobre o impacto da pandemia de COVID-19 na força de trabalho de enfermagem, como taxas crescentes de enfermeiros relatando esgotamento e deixando a profissão. Com base nos resultados de mais de 100 estudos, incluindo revisões sistemáticas, o relatório apontou que 40% a 80% dos enfermeiros experimentaram sintomas de sofrimento psicológico durante a pandemia.

Dentre esses estudos, um levantamento global realizado em 2022 revelou que 20% a 38% dos enfermeiros respondentes nos EUA, Reino Unido, Singapura, Japão, França, Brasil e Austrália manifestaram a intenção de deixar seus papéis atuais no cuidado direto ao paciente no próximo ano. O estresse, o esgotamento e as ausências ao trabalho foram apontados como sintomas do atual estado laboral e de saúde dos enfermeiros. O relatório discute o papel vital, porém perigoso, que os enfermeiros desempenharam durante a pandemia e destaca que, sem investimentos suficientes para apoiar esses profissionais, não haverá recuperação e reconstrução eficazes dos sistemas de saúde^(18)^.

A realidade descrita pelos participantes do presente estudo é semelhante à encontrada em outros estudos^(19,20)^, que também apontaram condições insalubres de atuação profissional durante a pandemia, enfatizando a ausência de EPIs adequados e/ou disponíveis em quantitativos suficientes, ausência de insumos básicos, número insuficiente de profissionais e longas jornadas de trabalho, sob acentuado estresse psicológico, e até mesmo o adoecimento físico e mental.

Os recursos humanos são um dos principais pilares para o alcance das metas de eliminação da TB. A relevância do profissional de enfermagem no desenvolvimento das ações de controle da doença traz à tona a iminente necessidade da recuperação e valorização da força de trabalho de enfermagem, o que exige estratégias e ações institucionais e governamentais urgentes, com formulação e implementação de políticas públicas de prevenção, contenção, diagnóstico e tratamento do adoecimento ocupacional^(17)^.

Os achados desta investigação demonstraram que as principais ações de controle da TB sofreram alterações, em virtude do remanejamento de recursos humanos para atuar na linha de frente contra a COVID-19, do distanciamento social e das mudanças na organização do fluxo de trabalho. Esses dados são corroborados por outros estudos^(21-23)^, que também identificaram quedas no desempenho das ações de controle da doença, em relação ao desempenho anterior à pandemia da COVID-19, em diversas localidades do Brasil e do mundo.

A redução da detecção de casos, em nível global, poderá refletir no aumento da mortalidade pela doença, afetando, principalmente, as pessoas mais vulneráveis^(21)^. No Brasil, observou-se redução dos casos confirmados notificados de TB pulmonar em todas as regiões, exceto no Norte, durante o período da pandemia^(24)^, dados esses que corroboram o estudo da *Global Tuberculosis Network*, que indicou que a taxa de diagnóstico de TB e de infecção latente diminuiu durante a pandemia de COVID-19 em muitos países^(25)^.

A busca ativa de SR, que foi interrompida no cenário estudado, é uma ação que contribui efetivamente para a detecção dos casos de TB e para o rápido início do tratamento, com o objetivo de interromper a cadeia de transmissão e reduzir a incidência da doença a longo prazo. A intensificação da busca ativa de casos, considerando as particularidades das populações mais vulneráveis nos territórios, é uma das principais estratégias do Plano Nacional pelo Fim da Tuberculose^(26)^.

A adesão ao tratamento é outro aspecto relevante para a prevenção e controle da doença, visto que a baixa adesão pode levar ao desenvolvimento de TB resistente aos medicamentos, além de favorecer a continuidade da cadeia de transmissão^(27)^. Uma das estratégias preconizadas pela OMS e pelo Ministério da Saúde para melhorar a adesão é a adoção do TDO^(26)^.

Estudos^(28-30)^ apontam os benefícios e a eficiência do TDO no controle da TB. Um deles, realizado em Curitiba-PR, mostrou que o TDO teve um papel fundamental no controle da doença, com redução do número de casos novos, diminuição da taxa de interrupção do tratamento e de óbitos por TB^(29)^. Tais evidências trazem à tona a relevância da retomada e reorganização desta estratégia para o enfrentamento da doença no período pós-pandêmico.

Os protocolos de atenção à saúde de Florianópolis foram citados como um ponto forte, que forneceu subsídios importantes para a organização interna do serviço no início da pandemia. Apesar disso, identificou-se que o protocolo municipal de atenção à TB não foi suficiente para manter a quantidade e qualidade das ações no contexto pandêmico.

A Coordenação-Geral de Vigilância das Doenças de Transmissão Respiratória recomendou, em março de 2020, a organização da rede de atenção à saúde local para que pessoas com sinais e sintomas de TB tivessem acesso aos serviços de saúde e aos exames laboratoriais. Recomendou, ainda, a organização da rede de saúde e a orientação do diagnóstico, reforçando a importância da triagem bilateral TB-COVID, além da utilização de estratégias tecnológicas disponíveis localmente para realizar o contato com o usuário^(31)^.

Contudo, houve dificuldade na efetivação dessas recomendações, principalmente por insuficiência de recursos humanos e materiais, além do intenso deslocamento do foco de atenção dos profissionais e dos órgãos de saúde para a COVID-19, o que levou a uma situação de invisibilidade da TB, principalmente no que se refere às ações de detecção.

A recomendação implementada de forma mais efetiva no cenário estudado foi a utilização de estratégias tecnológicas para o acompanhamento dos usuários do serviço, estratégias essas que também foram utilizadas, de forma exitosa, em outros locais do Brasil e do mundo^(32)^. Entende-se que as experiências vivenciadas apontam alguns caminhos interessantes para o uso futuro de tecnologias e telecomunicação na área da saúde, reforçando a necessidade de maiores investimentos em ciência e tecnologia e em estratégias de enfrentamento das iniquidades de acesso^(33)^.

Os prejuízos ocasionados pela pandemia reforçam a necessidade do compromisso programático institucional e governamental, do cuidado integral e longitudinal, com acesso continuado ao diagnóstico e tratamento, assim como a vigilância de agravos nos territórios, especialmente naqueles onde vivem/trabalham populações com maior vulnerabilidade. A APS tem um papel estratégico no enfrentamento da TB, a partir de estratégias de busca ativa de SR, diagnóstico precoce, tratamento oportuno e do acompanhamento por meio do TDO, visando aumentar as taxas de cura e reduzir a cadeia de transmissão e o surgimento de TB multidroga resistente^(34)^.

O fortalecimento da APS é condição indispensável para o avanço no controle da doença, visto que um acompanhamento ineficaz, por parte das equipes de APS, pode retardar o diagnóstico e favorecer a interrupção do tratamento. É necessário que os profissionais adotem uma atitude proativa, acolhedora e resolutiva no cuidado à pessoa com TB e sua família, considerando cada encontro como uma oportunidade para a elaboração ou continuidade de um projeto terapêutico colaborativo, onde o profissional e o serviço sejam corresponsáveis junto ao usuário^(35)^.

Assim, é necessária e urgente a reorganização da rede de atenção à TB, com a retomada das ações suspensas e/ou prejudicadas, valorização dos profissionais de saúde envolvidos nessas ações, fortalecimento das estratégias de prevenção, diagnóstico, tratamento e vigilância, além da atuação intersetorial integrada, para a superação dos impactos da pandemia e em prol dos compromissos assumidos na Estratégia *End TB* e nos ODS.

### Limitações do estudo

A coleta de dados foi realizada no final do período pandêmico (dezembro de 2022 a abril de 2023), de modo que alguns aspectos referentes à experiência profissional na pandemia podem ter sido esquecidos ou reelaborados pelos participantes. Outra limitação se refere à ausência de devolutiva das transcrições das entrevistas aos participantes, para comentários e/ou correções. Por fim, aponta-se que os dados provêm de um único cenário e talvez os resultados não possam ser generalizados para todas as populações ou contextos.

### Contribuições para a Área da Saúde e para a Enfermagem

O estudo contribui para a compreensão dos desafios enfrentados pelos profissionais da APS na oferta e realização de ações de controle da TB no contexto pandêmico, e aponta aspectos relevantes que devem ser priorizados na retomada e reorganização dessas ações. As políticas de saúde devem considerar as repercussões pandêmicas; partir da realidade atual vivenciada pelos atores (profissionais de saúde) que atuam diretamente nas ações de controle; e incorporar evidências científicas para reestruturar as estratégias de enfrentamento.

Os resultados desta pesquisa podem oferecer subsídios para a elaboração de políticas e estratégias voltadas à retomada e reorganização das ações de controle e eliminação da TB como problema de saúde pública, principalmente a nível municipal e estadual, assim como políticas voltadas ao fortalecimento e valorização da APS e da força de trabalho em saúde, com destaque para a enfermagem.

## CONSIDERAÇÕES FINAIS

Os achados evidenciaram a vulnerabilidade programática, materializada no despreparo e falta de recursos das diversas instâncias governamentais e dos serviços de saúde para lidar com a pandemia, enquanto situação de emergência em saúde pública, sem que houvesse graves prejuízos na oferta de serviços essenciais, como as ações de controle da TB. Soma-se a isso a falta de ações de monitoramento e cuidado referente à força de trabalho em saúde, principalmente os profissionais de enfermagem.

As metas ambiciosas dos ODS e da estratégia *End TB* exigem a implementação de ações e políticas de enfrentamento centradas na integralidade e intersetorialidade do cuidado, na disponibilidade de recursos materiais e humanos alocados de forma estratégica e eficaz, na valorização e recuperação da força de trabalho em saúde, que realiza as ações de controle da doença, e na intensificação do investimento em pesquisa e inovação, para que os desfechos favoráveis e o alcance das metas sejam possíveis. Esse é o caminho, resta seguir.
